# Epidemiology and grade 2 disability of leprosy among migrant and resident patients in Guangdong: an unignorable continued transmission of leprosy

**DOI:** 10.3389/fpubh.2023.1275010

**Published:** 2023-11-24

**Authors:** Lei Chen, Daocheng Zheng, Ming Li, Meiling Zeng, Bin Yang, Xiaohua Wang

**Affiliations:** Dermatology Hospital, Southern Medical University, Guangzhou, China

**Keywords:** leprosy, migrant, grade 2 disability, influencing factors, cross-sectional study

## Abstract

**Introduction:**

Leprosy remains a major public health concern worldwide and one of the leading causes of disability. New cases of leprosy with grade 2 disability (G2D) often reflect delayed detection due to the limited capacity of the health system to recognize leprosy early. This study aimed to describe the epidemiology and G2D of leprosy among migrant and resident patients with leprosy in Guangdong province, China.

**Methods:**

Data on newly diagnosed cases of leprosy were collected from the leprosy management information system in China. Descriptive statistical analysis was used to describe the status of G2D. Joinpoint regression model and logistic regression were performed to analyze the temporal trends and influencing factors for G2D.

**Results:**

The G2D rate among migrant, resident, and total patients with leprosy was 17.5%, 18.7%, and 18.4%, respectively. The total G2D rate increased significantly from 18.0% in 2001 to 25.7% in 2021 (average annual per cent change: 2.5%). Multivariate analysis revealed that factors that negatively influence G2D between migrant and resident patients included delayed discovery time (migrants: OR = 2.57; residents: OR = 4.99) and nerve damage when diagnosed (migrants: OR = 9.40; residents: OR = 21.28).

**Discussion:**

Our findings indicate that the targeted intervention measures implemented by our health system are urgently needed to improve the current situation, such as programs to promote early detection, strengthen awareness and skills of healthcare workers, and rehabilitation for disabled patients to improve their quality of life.

## 1 Introduction

Hansen’s disease or leprosy is a neglected tropical disease (NTD) mainly caused by *Mycobacterium leprae*, a slow-growing *Mycobacterium* discovered by Hansen, which predominantly affects the skin, peripheral nerves, mucous membranes, liver, and kidney (1, 2). In 2008, *Mycobacterium lepromatosis* was identified as a new species and the second causal agent of leprosy. However, *M. lepromatosis* has been implicated in a small number of leprosy cases, and the clinical aspects of leprosy caused by *M. lepromatosis* are poorly characterized (3). Since 1982, the World Health Organization (WHO) has recommended multi-drug therapy (MDT), which has been used to treat more than 15 million patients in the last 30 years and has made leprosy a curable disease (4).

Although MDT has reduced the number of cases in treatment, it had less impact on the number of new cases. Despite the availability of health facilities, there continue to be barriers to early diagnosis and treatment of leprosy. The lack of awareness of the disease among the general population and many consultations—suggestive of ill-trained health staff in suspecting and managing a leprosy case—were associated with a longer diagnostic delay, thus contributing to the ongoing transmission and disability-related disease burden (5). There were 202,185 new cases of leprosy worldwide, with a new case detection rate of 25.9 per million population in 2019 (6). In China, according to statistics released by the National Center for Leprosy Control, a total of 521 new cases of leprosy were detected, with a new case detection rate of 0.037 per 100,000 population in 2018 (7).

Guangdong province is the most severe leprosy epidemic area, with the highest number of cases in history; records showed that 96,797 people were affected by leprosy by 2020, representing approximately one-fifth of all leprosy patients in China. Regarding the new cases reported in 2018, Guangdong province ranked third in the number of new cases reported (57 cases), trailing Yunnan (174 cases) and Guizhou province (61 cases). These three provinces accounted for 56.0% of newly detected cases in China (7). The epidemic has the potential to be underestimated. There may be cases of oligosymptomatic leprosy with subclinical infection without diagnosis. This underdiagnosis may lead to underreporting to the leprosy surveillance system.

As the first province in China in terms of floating population and GDP, Guangdong province has great attraction and development potential in household registration immigration. According to the Guangdong Statistical Yearbook 2021, approximately 28.15 million migrants live in Guangdong province, representing 22.3% of the total permanent population (8). Movement can be a strategy to achieve a higher standard of living and access to better employment, education, and health service infrastructure, mainly among resource-poor rural areas and urban centers (9). Migration can lead to changes in circumstances that influence the conditions and risks associated with disease transmission, particularly among the poor, who are disproportionately affected (9, 10). Therefore, migration has been identified as one of the social determinants that influence the transmission dynamics of NTDs, including leprosy, which was identified as an infection risk factor associated with poverty (11, 12).

To improve our understanding of the epidemic situation of leprosy between migrant and resident populations, we detailed the demographic, clinical, geographic characteristics, and temporal trend of patients with leprosy in Guangdong province, China, 2001–2021. We also evaluated the status of grade 2 disability (G2D) and its associated influencing factors among migrant and resident patients with leprosy.

## 2 Methods

### 2.1 Data sources and diagnosis standard

Information on newly diagnosed leprosy cases were collected from the leprosy management information system (LEPMIS) in China. LEPMIS was designed by the Chinese government in 2010 and reports data on new, recurrent, and prevalent cases of leprosy (13). LEPMIS includes not only related information on disease discovery, diagnosis, and treatment, but also information on all aspects of leprosy management, including basic demographic information, source of infection, close contacts, follow-up after cure, etc. Therefore, LEPMIS was a detailed and comprehensive database that achieved lifelong leprosy cases and even contained information on death. Data from patients with leprosy from 2001 to 2021 were collected from paper files and uploaded to LEPMIS by staff from municipal, county, and district-level professional leprosy prevention and control institutions. Oral consent was obtained from each participant before the interview and each participant could decline to participate in this survey at any step.

The diagnosis of new leprosy was based on the Leprosy Diagnosis Standard WS291-2018 (14). Patients with clinical manifestations (skin lesions or peripheral nerve lesions) and positive laboratory tests for leprosy (skin smear test for bacteria or histological examination) were diagnosed as confirmed cases. Disability classification was based on the Disability Classification Standard for Leprosy, WHO, 1998 (15). In 1988, the WHO Expert Committee on Leprosy substantially simplified the disability grading system into a three-grade (0, 1, and 2) classification system (16). Patients with G2D have visible deformities (i.e., hand ulcers, absorption or contractures of the digits, plantar ulcers, callosities, foot drop, or claw) or severe visual impairment (i.e., cannot read the fingers at a distance of 6 m). Patients with grade 1 disability have loss of sensation or eye problems (irregular blinking) due to the presence of leprosy, but no visible deformities, including muscle weakness without clawing. Nerve involvement in leprosy is considered to occur when there are signs of pain or nerve thickening on palpation of the nerves, when there is loss of sensation according to the monofilament test, or when motor impairment is observed (17). Patients with grade 0 disability have no loss of sensation, no visible deformity, and no eye problems due to leprosy. In 1997, the eighth report of the WHO Expert Committee on Leprosy endorsed this grading system with the amendment that lagophthalmos, iridocyclitis, and corneal opacities should be included in the grade 2 criteria (18).

### 2.2 Measures

Migrant patients with leprosy were defined as patients with leprosy who live in other places for work or live outside the county or municipal district where their registered permanent residence is located. The assignment of independent variables in the multivariate analysis was as follows: (1) age: 1, <30; 2, 30–44; 3, 45–59; 4, ≥60. (2) Sex: 1, female; 2, male. (3) Nationality: 1, others; 2, Han. (4) Occupation: 1, students; 2, workers or office staff; 3, unemployment or retiree; 4, farmers or herders. (5) Marriage status: 1, unmarried; 2, married; 3, divorced or widowed. (6) Delayed discovery time: 1, ≤24 months; 2, >24 months. (7) Skin lesion when diagnosed: 1, ≤5; ≥6. (8) Leprosy reaction: 1, no; 2, yes. (9) Nervous lesions when diagnosed: 1, 0; 2, 1; 3, ≥2. (10) Treatment classification: 1, paucibacillary (PB), including indeterminate (I), tuberculoid (TT), and borderline-tuberculoid (BT) cases with negative skin smear test for bacteria, skin lesions ≤5 and nerve lesion ≤1; 2, multibacillary (MB), including lepromatous (LL), borderline-lepromatous (BL), and mid-borderline (BB) cases with positive skin smear test for bacteria, or cases with negative skin smear test for bacteria but skin lesions ≥6 or nerve lesions ≥2, or cases with lesions ≥1 and also have characteristics of size, sensory, or morphological losses of advanced leprosy patients.

### 2.3 Statistical methods

A newly diagnosed leprosy case database was established in Excel 2010 (Microsoft Corp., Redmond, WA, United States), and the data were cleaned by logical error detection. Statistical analyses, including descriptive statistical analysis, chi-square test, bivariate and multiple logistic regression analysis, were performed using IBM SPSS Statistics 24.0 (IBM Corp., Armonk, NY, United States). Basic descriptive analyses, including frequency, percentage, and mean (SD), were used to describe demographic and clinical characteristics. Geographic distribution analysis was performed using MapInfo Professional version 11.0 (Pitney Bowes Software Inc.). Joinpoint regression analysis was performed using the Joinpoint Regression Program version 4.9.1.0 (Statistical Research and Applications Branch, National Cancer Institute).

### 2.4 Ethics statement

No personally identifiable information was included in the database used for the analysis. All data included in the study were stored on a removable hard disk and unauthorized access to them was not possible.

## 3 Results

### 3.1 Demographic and clinical characteristics of leprosy from 2001 to 2021

**Figure 1:**
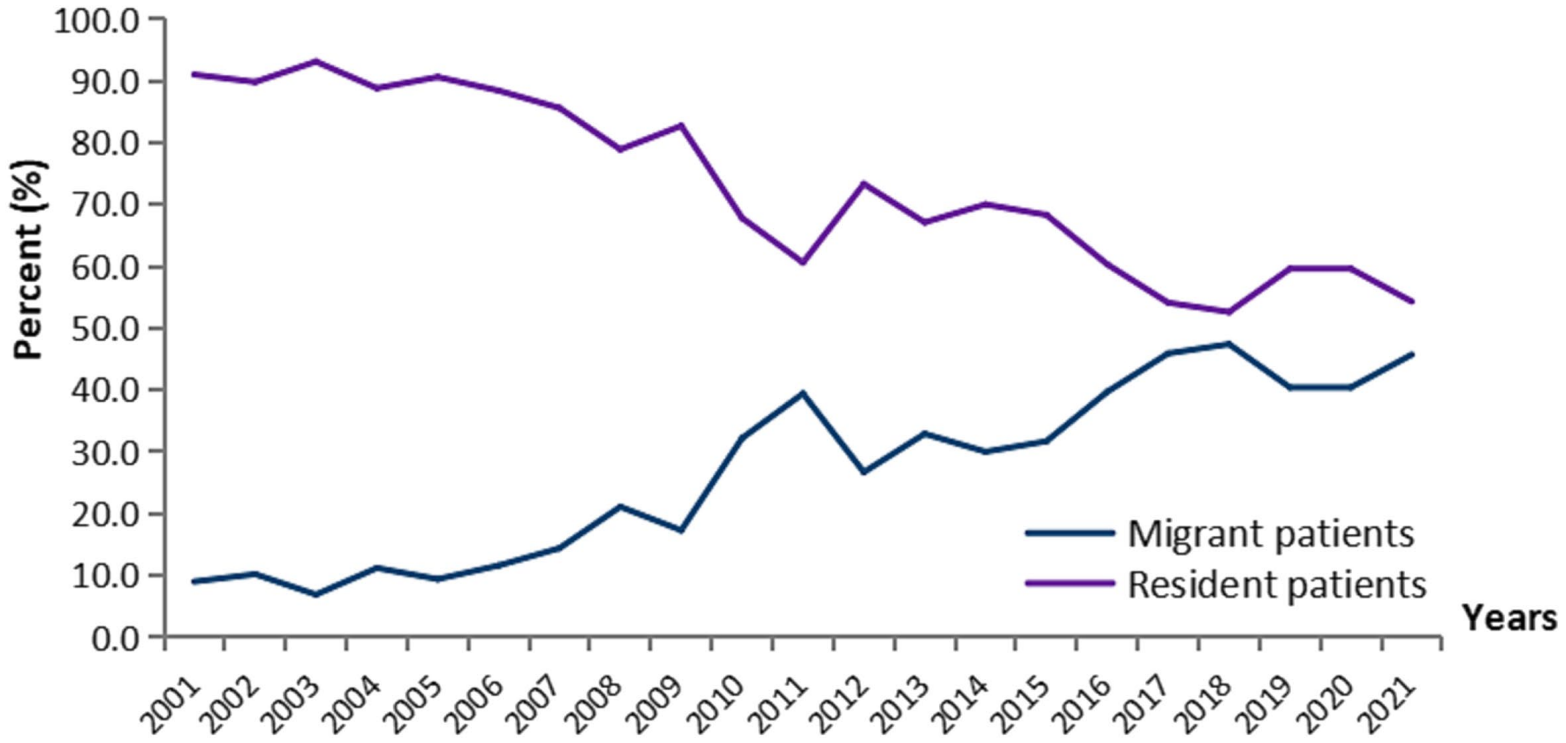
Trends of migrant and resident patients with leprosy in Guangdong province, China, 2001–2021.

Of the 1980 newly diagnosed cases of leprosy registered during the study period, 452 (22.8%) were migrant patients, while 1,528 (77.2%) were resident patients. The proportion of migrant cases increased from 9.0% in 2001 to 45.7% in 2021 in Guangdong, China (Figure 1). Among the 452 migrant patients, 328 from other provinces were relatively close in location [top five provinces: Hunan (74), Guizhou (55), Sichuan (48), Jiangxi (38), and Guangxi (34)], 116 from other areas of Guangdong, four from Hongkong, three from Indonesia, and one from Mali. Among 1,980 patients with leprosy, 1,302 (65.8%) were males. The vast majority (94.9%) were Han nationality and 1,105 cases were farmers or herders, representing 58.7%. Between 2001 and 2021, the average age of newly diagnosed patients with leprosy in Guangdong was 43.1 (SD = 18.0) years. During this period, 51 cases of pediatric patients (aged <14 years) were detected, with a male-to-female ratio of 1:3 (Table 1).

**Table 1:** Demographic and clinical characteristics of migrant and resident patients with leprosy in Guangdong, China, 2001–2021 (*N* = 1,980).

	Migrant patients (*n* = 452)		Resident patients (*n* = 1,528)		Total		Chi-square	*p*-value
Characteristics	Frequency	Percentage (%)	Frequency	Percentage (%)	Frequency	Percentage (%)		
**Age (*n* = 1,978)**							136.837	<0.001
<30	165	36.5	399	26.1	564	28.5		
30–44	180	39.8	319	20.9	499	25.2		
45–59	80	17.0	437	28.6	517	26.1		
≥60	27	6.0	371	24.3	398	20.1		
Mean (SD)	36.5 (12.9)		45.1 (18.8)		43.1 (18.0)			
**Sex (*n* = 1,980)**							8.459	0.002
Female	129	28.5	549	35.9	678	34.2		
Male	323	71.5	979	64.1	1,302	65.8		
**Nationality (*n* = 1,980)**							216.452	<0.001
Others	83	18.4	17	1.1	100	5.1		
Han	369	81.6	1,511	98.9	1,880	94.9		
**Occupation (*n* = 1,881)**							506.260	<0.001
Students	11	2.6	115	7.9	126	6.7		
Workers or office staff	284	67.1	199	13.6	483	25.7		
Unemployment or retiree	36	8.5	131	9.0	167	8.9		
Farmers or herders	92	21.7	1,013	69.5	1,105	58.7		
**Education (*n* = 1,314)**							103.801	<0.001
Primary schools or lower	89	22.3	455	49.7	544	41.4		
Junior high school	183	45.9	334	36.5	517	39.3		
High school	99	24.8	100	10.9	199	15.1		
University or higher	28	7.0	26	2.8	54	4.1		
**Marriage (*n* = 1,464)**							4.444	0.111
Unmarried	127	28.5	241	23.7	368	25.1		
Married	307	68.8	739	72.6	1,046	71.4		
Divorced or widowed	12	2.7	38	3.7	50	3.4		
**Delayed discovery time (*n* = 1,980)**							10.515	0.001
≤24 months	242	53.5	948	62.0	1,190	60.1		
>24 months	210	46.5	580	38.0	790	39.9		
Mean (SD) (months)	36.8 (48.5)		31.4 (43.7)		32.6 (44.9)			
**Case detection (*n* = 1,980)**							0.131	0.727
Dermatological consultation	374	82.7	1,253	82.0	1,627	82.2		
Others (contact, epidemic point investigation, self-reporting, mutual reporting, or census)	78	17.3	275	18.0	353	17.8		
**Source of infection (*n* = 355)**							0.706	0.433
Outside family transmission	25	27.5	85	32.2	110	31.0		
Intra-household transmission	66	72.5	179	67.8	245	69.0		
**Skin lesion when diagnosed (*n* = 1,963)**							23.391	<0.001
≤5	107	23.7	542	35.9	649	33.1		
≥6	345	76.3	969	64.1	1,314	66.9		
**Leprosy reaction (*n* = 1,945)**							2.186	0.144
No	342	76.7	1,198	79.9	1,540	79.2		
Yes	104	23.3	301	20.1	405	20.8		
**Nervous lesion when diagnosed (*n* = 1,964)**							9.339	0.009
0	169	37.6	469	31.0	638	32.5		
1	71	15.8	217	14.3	288	14.7		
≥2	210	46.7	828	54.7	1,038	52.9		
L**esions when diagnosed (*n* = 1,955)**							13.718	0.002
No lesion	0	0.0	3	0.2	3	0.2		
Only skin lesion	169	37.6	462	30.7	631	32.3		
Only nervous lesion	0	0.0	19	1.3	19	1.0		
Both skin and nervous lesions	281	62.4	1,021	67.8	1,302	66.6		
**Case classification (*n* = 1,470)**							36.756	<0.001
Laboratory diagnosed cases	431	97.1	889	86.6	1,320	89.8		
Clinically diagnosed cases	13	2.9	137	13.4	150	10.2		
**Bacterial index (BI) (*n* = 1,837)**							21.547	<0.001
0	112	26.2	438	31.1	550	29.9		
0–4	200	46.7	731	51.9	931	50.7		
≥4	116	27.1	240	17.0	356	19.4		
**Ridley–Jopling classification/clinic form** ^a^ **(*n* = 1,958)**							9.158	0.057
TT	34	7.6	160	10.6	194	9.9		
BT	112	25.1	385	25.5	497	25.4		
BB	48	10.7	163	10.8	211	10.8		
BL	113	25.3	424	28.1	537	27.4		
LL	140	31.3	379	25.1	519	26.5		
**Treatment classification** ^b^ **(*n* = 1,980)**							2.453	0.117
PB	45	10.0	117	7.7	162	8.2		
MB	407	90.0	1,411	92.3	1,818	91.8		
**Disability (*n* = 1,980)**							0.356	0.581
Others	373	82.5	1,242	81.3	1,615	81.6		
G2D^c^	79	17.5	286	18.7	365	18.4		

a TT, tuberculoid; BT, borderline-tuberculoid; BB, mid-borderline; BL, borderline-lepromatous; LL, lepromatous.

b PB, paucibacillary; MB, multibacillary.

c G2D, grade 2 disability.

A total of 1,818 patients were diagnosed with MB leprosy, which represents 91.8%. According to Ridley–Jopling classification, patients with leprosy were more likely to be classified as having BL (537 patients, 27.4%), LL (519 patients, 26.5%), and BT (497 patients, 25.4%). Most patients have both skin lesions and nervous lesions (1,302 patients, 66.6%), and a positive skin smear test for bacteria [bacterial index (BI) >0: 1,287 patients, 70.1%] when diagnosed.

The results of the chi-square test showed that there was a significant difference between migrant and resident patients from different groups of age, sex, nationality, occupation, education, delayed discovery time, skin lesions when diagnosed, nervous lesions when diagnosed, case classification, and BI (*p* < 0.05) (Table 1).

### 3.2 Geographic and temporal trend of G2D from 2001 to 2021

**Figure 2:**
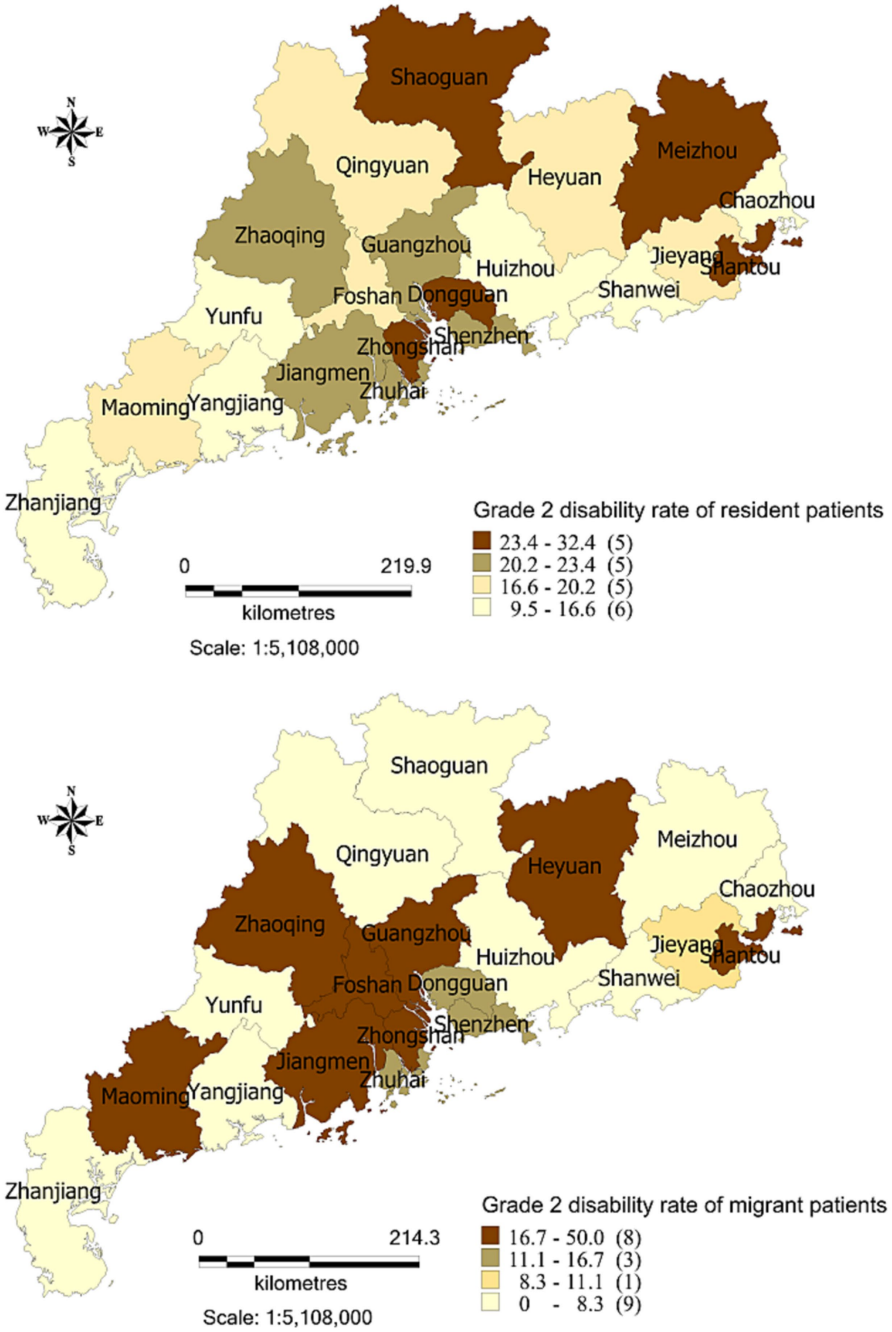
Geography distribution of the grade 2 disability rate between migrant and resident patients with leprosy in Guangdong province, China, 2001–2021.

The G2D rate of newly diagnosed migrant leprosy patients was 17.5% between 2001 and 2021, ranging from 0 (Chaozhou and the other six cities) to 50.0% in Heyuan (northern Guangdong) (Figure 2). In contrast, the rate of G2D of newly diagnosed resident patients with leprosy was 18.7 between 2001 and 2021, ranging from 9.5% in Chaozhou (eastern Guangdong) to 32.4% in Shaoguan (northern Guangdong) (Figure 2).

Throughout the 21-year period, the G2D rate increased significantly over time, from 18.0% in 2001 to 25.7% in 2021 [average annual per cent change (AAPC) 2.5, 95% CI: 1.0–3.9]. Among 1,528 resident patients, 286 cases of leprosy with G2D were reported; the G2D rate also increased significantly over time from 12.7% in 2005 to 36.8% in 2021 (AAPC 5.0, 95% CI: 3.5–6.6) (Table 2).

**Table 2:** Joinpoint regression analysis of the trend for grade 2 disability rate among migrant and resident leprosy patients in Guangdong province, China, 2001–2021 (*N* = 1,980).

	Grade 2 disability rate (%) (2001–2021)	AAPC^a^ (95% CI)	*p*-value
**All patients with leprosy**	18.0–25.7	2.5 (1.0**–**3.9)	0.002
**Migrant patients with leprosy (*n* = 452)**	27.3–12.5	9.7 (−7.0**–**29.3)	0.255
**Resident patients with leprosy (*n* = 1,528)**	17.1–36.8	3.3 (0.8**–**5.9)	0.010
Segment 2001–2005	17.1–12.7	−3.3 (−14.2**–**9.1)	0.564
Segment 2005–2021	12.7–36.8	5.0 (3.5**–**6.6)	<0.001

a AAPC, average annual percent change.

### 3.3 Bivariate and multiple logistic regression analysis of G2D

Univariate logistic regression analysis showed that age (≥60, migrant: OR = 3.50, 95% CI: 1.24–9.89; resident: OR = 2.25, 95% CI: 1.56–3.24), delayed discovery time (>24 months, migrant: OR = 3.46, 95% CI: 2.04–5.87; resident: OR = 3.78, 95% CI: 2.89–4.95), nerve lesions when diagnosed (≥2, migrant: OR = 11.38, 95% CI:4.78–27.08; resident: OR = 15.73, 95% CI: 8.70–28.44), and treatment classification (MB, migrant: OR = 10.42, 95% CI: 1.41–76.88; resident: OR = 7.06, 95% CI: 2.58–19.29) were the influencing factors for G2D for both migrant and resident patients with leprosy (Table 3).

**Table 3:** Bivariate and multiple logistic regression analysis of grade 2 disability among migrant and resident patients with leprosy in Guangdong province, China, 2001–2021 (*N* = 1,980).

Bivariate logistic regression										
Characteristics	Migrant patients with leprosy (*n* = 452)					Resident patients with leprosy (*n* = 1,528)				
	Grade 2 disability rate (%)	*p*-value	OR	95% CI		Grade 2 disability rate (%)	*p*-value	OR	95% CI	
**Age**										
<30	17.0	0.367	1.43	0.66	3.11	13.8		1.00		
30–44	17.8	0.288	1.51	0.70	3.25	15.0	0.632	1.11	0.73	1.68
45–59	12.5		1.00			19.5	0.029	1.51	1.04	2.19
≥60	33.3	0.018	3.50	1.24	9.89	26.4	<0.001	2.25	1.56	3.24
**Sex**										
Female	15.50		1.00			12.8		1.00		
Male	18.3	0.485	1.22	0.70	2.12	22.1	<0.001	1.94	1.45	2.60
**Nationality**										
Others	9.6		1.00			5.9		1.00		
Han	19.2	0.042	2.23	1.03	4.84	18.9	0.203	3.72	0.49	28.16
**Occupation**										
Students	18.2	0.727	1.38	0.23	8.34	8.7		1.00		
Workers or office staff	15.8	0.761	1.17	0.43	3.16	11.1	0.506	1.31	0.60	2.86
Unemployment or retiree	13.9		1.00			13.7	0.217	1.67	0.74	3.79
Farmers or herders	21.7	0.318	1.72	0.59	5.00	21.9	0.001	2.95	1.52	5.73
**Marriage**										
Unmarried	18.9	0.457	1.23	0.72	2.10	14.9		1.00		
Married	16.0		1.00			22.7	0.010	1.68	1.13	2.48
Divorced or widowed	33.3	0.126	2.63	0.76	9.08	15.8	0.891	1.07	0.42	2.74
**Delayed discovery time**										
≤24 months	9.5		1.00			10.9		1.00		
>24 months	26.7	<0.001	3.46	2.04	5.87	31.6	<0.001	3.78	2.89	4.95
**Skin lesion when diagnosed**										
≤5	12.1		1.00			12.9		1.00		
≥6	19.1	0.100	1.71	0.90	3.24	22.2	<0.001	1.92	1.43	2.58
**Leprosy reaction**										
No	15.2		1.00			17.40		1.00		
Yes	26.0	0.013	1.96	1.15	3.32	23.90	0.010	1.49	1.10	2.02
**Nervous lesion when diagnosed**										
0	3.6		1.00			2.60		1.00		
1	14.1	0.005	4.45	1.55	12.78	14.7	<0.001	6.59	3.32	13.07
≥2	29.5	<0.001	11.38	4.78	27.08	29.2	<0.001	15.73	8.70	28.44
**Treatment classification** ^a^										
PB	2.2		1.00			3.4		1.00		
MB	19.2	0.021	10.42	1.41	76.88	20.0	<0.001	7.06	2.58	19.29

Multiple logistic regression														
Characteristics	Migrant leprosy patients (*n* = 452)							Resident leprosy patients (*n* = 1,528)						
	Regression coefficient	Standard error	Wald *χ*^2^	*p*-value	OR	95% CI		Regression coefficient	Standard error	Wald *χ*^2^	*p*-value	OR	95% CI	
**Age**														
<30	—							Ref						
30–44	—	—	—	—	—	—	—	0.185	0.335	0.305	0.581	1.20	0.62	2.32
45–59	—	—	—	—	—	—	—	0.97	0.284	11.672	0.001	2.64	1.51	4.60
≥60	—	—	—	—	—	—	—	1.278	0.286	20.026	<0.001	3.59	2.05	6.28
**Sex**														
Female	—							Ref						
Male	—	—	—	—	—	—	—	0.8	0.222	12.987	<0.001	2.23	1.44	3.44
**Delayed discovery time**														
≤24 months	Ref							Ref						
>24 months	0.942	0.283	11.108	0.001	2.57	1.47	4.47	1.608	0.203	62.492	<0.001	4.99	3.35	7.43
**Nervous lesion when diagnosed**														
0	Ref							Ref						
1	1.438	0.542	7.030	0.008	4.21	1.46	12.19	2.227	0.536	17.273	<0.001	9.27	3.24	26.50
≥2	2.241	0.447	25.133	<0.001	9.40	3.91	22.57	3.058	0.470	42.307	<0.001	21.28	8.47	53.48

a PB, paucibacillary; MB, multibacillary.

Multivariate analysis revealed that factors that influence G2D in migrant patients with leprosy were the delayed discovery time (>24 months, OR = 2.57, 95% CI: 1.47–4.47), and nerve damage when diagnosed (≥2, OR = 9.40, 95% CI: 3.91–22.57). Meanwhile, in resident patients with leprosy, the influencing factors for G2D were age [≥60, OR = 3.59, 95% CI: 2.05–6.28, sex (male, OR = 2.23, 95% CI: 1.44–3.44)], delayed discovery time (>24 months, OR = 4.99, 95% CI: 2.35–7.43), and nerve lesion when diagnosed (≥2, OR = 21.28, 95% CI: 8.47–53.48) (Table 3).

## 4 Discussion

With the worldwide implementation of WHO MDT in the 1980s, the global burden of leprosy has decreased. However, a certain number of new patients with leprosy with G2D are still found when diagnosed, reflecting a failure in early detection of leprosy and indicating that transmission continues (19). Of the 121 countries that submitted data to the WHO on G2D in 2020, 68 countries reported 7,198 new G2D cases, which represents 5.7% of 127,396 new cases of leprosy worldwide (20). Although leprosy has a steadily low prevalence in China, the rate of G2D among new cases of leprosy was 17.7% in 2020, while our research showed that the rate of G2D was 18.4% in Guangdong province, China, between 2001 and 2021. Although leprosy cases with G2D have been diagnosed and reported, cases of oligosymptomatic leprosy with subclinical infection can easily be overlooked. Therefore, it leads to underdiagnosis, underreporting to the surveillance system, and an underestimation of the leprosy epidemic.

Leprosy remains one of the leading causes of deformity and physical disability. Our study showed the same influencing factors for G2D among migrant and resident patients with leprosy, including delayed discovery and nervous lesions when diagnosed. The burden of leprosy G2D in new cases often indicates a delayed detection, often due to a lack of awareness in the community of early signs of leprosy, a delay in seeking care, or the limited capacity of the health system to recognize leprosy early (20). Srinivas et al. (21) found that delayed diagnosis is a major challenge in the leprosy program in India. Patient delay and healthcare provider delay have been significant risk factors for disability among adult cases of leprosy. Moreover, since a large proportion of cases with disabilities ignored initial symptoms as they believed that symptoms would disappear by themselves, patient delay became the main reason for the risk of disability.

Nervous lesions when diagnosed were another influencing factor for G2D among migrant and resident patients with leprosy. Due to bacterial proliferation or the immunologic response of the host to these bacilli or both, there is some degree of irreversible peripheral nerve damage in virtually all patients with leprosy. If not treated effectively, widespread destruction of the mixed peripheral nerve trunks can result in widespread skin anesthesia and widespread permanent muscle paralysis that affects the face, hands, and feet (22). Meanwhile, peripheral nerve trunks remove the sensation of pain. The lack of pain feedback allows patients with leprosy to damage and deform themselves. Most of the deformities attributed to leprosy are caused by this secondary damage (1).

Of the 1,980 cases of leprosy registered during the study period, 452 (22.8%) were migrant patients. Similarly to our previous research, the migrant cases originated mainly from two areas: the provinces surrounding Guangdong (Hunan and Jiangxi) and the southwestern provinces (Guizhou, Sichuan, and Guangxi), which have the highest incidence of leprosy in China (23). Due to the long incubation period of leprosy and the failure of current testing method to detect leprosy early, these migrants were identified and treated only after they migrated to Guangdong province, thus resulted in continued transmission between provinces in Chinese territory. We also found that the G2D rate among migrant patients with leprosy was 17.5%, which is related to delayed discovery and nervous lesions. Early diagnosis of leprosy can lead to breaking the chain of transmission and reducing the number of G2D cases (24, 25). Thus, the key point of resolve epidemiological problem is to fix the inability to diagnose in early period of leprosy. However, an accurate and timely diagnosis of the disease is still a challenge (26). Skin slit smear acid-fast staining is rapid and economic but has very low sensitivity and specificity. Definitive diagnosis of leprosy by clinic and pathological features requires experienced physicians. Quantitative polymerase chain reaction (qPCR) holds promise as a simple and sensitive diagnostic tool, but the infrastructure, such as equipment and trained professionals, is still a barrier to implementing qPCR in resource-limited settings (27). Early diagnosis and followed by effective treatment is crucial to avoiding continued transmission and controlling the disease, the need for more accurate tools for the detection and confirmation of leprosy among suspected individuals and more effective treatment regimens are indeed urgent.

Our study also found that all migrant patients with leprosy had skin or nervous lesion when diagnosed, nearly one quarter had leprosy reactions, and about one fifth had visible deformities. The pronounced damage and permanent disabilities frequently caused patients with leprosy to suffer stigma and prejudice. The management of migrant patients with leprosy has been an important issue for current leprosy prevention and control. Possible reasons for this are that patients with leprosy may fear discrimination to seek medical care. Otherwise, changing job and address frequently reduced the convenience to professional leprosy prevention and control institutions for treatment because of more time and transportation cost.

There are some limitations to this study. First, this study is a cross-sectional study, therefore, we were unable to draw causal inferences. Second, the clinical histories of some participants were incomplete due to the loss of records throughout the 21 years period, and the information of treatment classification was collected from LEPMIS which followed the instruction of WHO-MDT classification of leprosy treatment and the Leprosy Diagnosis Standard WS291-2018 in China, which may outdated and reduces the complexity of the disease. Third, although patients claim that their current address is the place where they live, work, and receive treatment, we cannot exclude individuals who work only in places other than their municipality as migrants. Furthermore, it is not known whether the infection occurred at the place of primary residence or at the present address due to the long incubation period of leprosy.

To reduce delay and promote early diagnosis, we have implemented leprosy symptom monitoring programs in Guangdong province since 2017. We propose eight early symptoms of leprosy. If doctors in medical institutions find suspected cases of leprosy, the cases can be referred to a local leprosy-designated diagnosis and treatment center for professional diagnosis and treatment through the “Guangdong Suspected Leprosy Symptom Monitoring System.” However, this is not enough; early case detection campaigns, such as active surveys in endemic areas, should be carried out periodically (21). Furthermore, continuing education programs for healthcare workers and the community should also place greater emphasis on raising awareness of the disease (19). Meanwhile, disability prevention and rehabilitation, even after effective treatment, remain essential for patients with leprosy with deformity to improve their quality of life, which is why we plan to establish a provincial-level leprosy correction surgery and rehabilitation center in the future.

## Data availability statement

The data analyzed in this study is subject to the following licenses/restrictions: Leprosy Control Department of Dermatology Hospital, Southern Medical University. Requests to access these datasets should be directed to XW, Wxh_21773@163.com.

## Ethics statement

The studies involving humans were approved by Medical Ethics Committee of Dermatology Hospital of Southern Medical University. The studies were conducted in accordance with the local legislation and institutional requirements. Written informed consent for participation in this study was provided by the participants’ legal guardians/next of kin.

## Author contributions

LC: Writing – original draft, Data curation. DZ: Writing – review & editing. ML: Writing – review & editing. MZ: Writing – review & editing. BY: Writing – review & editing. XW: Conceptualization, Funding acquisition, Writing – review & editing.
